# Long-term real-world data of ustekinumab in ulcerative colitis: the Stockholm Ustekinumab Study (STOCUSTE)

**DOI:** 10.1097/MEG.0000000000002854

**Published:** 2024-09-20

**Authors:** Haider Sabhan, Francesca Bello, Samer Muhsen, Alexandra Borin, Fredrik Johansson, Charlotte Höög, Ole Forsberg, Christina Wennerström, Mikael Lördal, Sven Almer, Charlotte Söderman

**Affiliations:** a Gastroenterology Unit, Medical Department, Capio St Göran Hospital; b Division of Gastroenterology, Medical Department, Karolinska University Hospital, Stockholm; c Department of Medicine, Division of Gastroenterology and Hepatology, Danderyd Hospital, Danderyd; d Department of Medicine, South Hospital, Stockholm; e Medical Library at Danderyd Hospital, Danderyd; f Department of Medicine, Karolinska Institutet, Huddinge; g Janssen Cilag AB; h Department of Medicine, Karolinska Institutet, Solna, Stockholm, Sweden

**Keywords:** long-, term follow-, up, real-, world data, ulcerative colitis, ustekinumab

## Abstract

**Background:**

Ustekinumab (UST) is an anti-interleukin-12/23 antibody used in the treatment of inflammatory bowel disease. This study includes patients treated at four hospitals in Stockholm to provide long-term real-world data.

**Methods:**

Retrospective study including patients diagnosed with ulcerative colitis and treated with UST between the years 2019 and 2021. Patients were followed until withdrawal of treatment, or until a predefined end of study, 31 July 2021. Disease activity was assessed with Physician Global Assessment (PGA); Ulcerative Colitis Endoscopic Index of Severity (UCEIS), laboratory parameters, and drug persistence. The primary outcome was steroid-free remission (PGA = 0) and response (decrease PGA ≥ 1 from baseline) at 3 and 12 months, respectively.

**Results:**

A total of 96 patients, 44 women and 52 men were included. The patients had either extensive colitis (69%), left-sided colitis (29%), or proctitis (3%). All but two patients were anti-TNF-experienced; 94 (98%) had failed ≥1, 59 (61%) ≥ 2, and 34 (35%) had failed ≥ 3 anti-TNF drugs. In addition, 28 (29%) had failed vedolizumab. At inclusion, 92/96 patients (96%) had active disease and four patients were in remission. Among patients who were treated with UST, 9/71 (13%) were in steroid-free remission at 3 months, and 26/33 (78%) were at 12 months. Withdrawal rates at 3 and 12 months, were 12 and 26%, respectively, mainly due to persisting disease activity (20%).

**Conclusion:**

In this group of patients with difficult-to-treat ulcerative colitis, UST was shown to be effective in the majority, with high drug persistence at 12 months in combination with a favorable safety profile.

## Introduction

Ulcerative colitis (UC) is a chronic inflammatory disease of the large intestine, which affects one in 400 people with a rising global incidence and prevalence. While most patients have a mild–moderate course, about 10–15% experience severe disease that usually requires hospitalization, immunosuppressive therapies, and corticosteroids, with a negative impact on health-related quality of life [1–3]. During the last 20 years, the need for surgery in UC patients has rapidly declined [4]. In addition, morbidity and mortality associated with the disease have also decreased significantly [4,5].

Although treatment of inflammatory bowel disease (IBD) has significantly improved with the introduction of monoclonal antibodies against tumor necrosis factor (anti-TNF) and α4β7 integrin (vedolizumab), a considerable proportion of patients either fail to respond or lose response over time to these agents [6]. This group of patients with difficult-to-treat disease needs other treatment options with different modes of action, to reach symptom relief or remission [7–10]. At present, ustekinumab (UST), JAK inhibitors, and ozanimod are available in clinical practice [11–13].

UST is a recombinant, human mAb to interleukins (ILs) 12 and 23. The targets the p40 subunit of IL-12 and IL-23, naturally occurring proteins that regulate the immune system and immune-mediated inflammatory disorders in the human body. UST was approved in 2016 for the treatment of moderate-to-severe Crohn’s disease following phase III randomized controlled trials (RCTs), and for UC in 2019, by the US Food and Drug Administration (FDA) [7,8]. In addition, the efficacy of UST has also been demonstrated in long-term extensions of RCTs and in post-hoc endoscopic outcome analysis [7–12]. A more generalizable knowledge of UST would be gained in a real-world setting with the important complement to the knowledge gained from the RCTs and to provide the necessary data needed in the drug application to support a fully comprehensive assessment [13,14].

The aim of this retrospective long-term cohort study was to evaluate clinical response with emphasis on steroid-free remission, drug persistence, and the need for additional medical treatment, to demonstrate the real-world experience of UST.

## Patients and methods

### Study population

A total of 96 adult patients with UC and treated with UST, at one of the four major hospitals in Stockholm, Sweden (Capio St Göran Hospital, Karolinska University Hospital, Danderyd Hospital, and South Hospital), were included in the study.

### Inclusion criteria

Inclusion criteria included adult patients (>18 years) with active UC. Every patient received at least the intravenous induction dose of UST since its approval in 2019. A few patients (*n* = 4) with previously severe diseases were included as they had been brought into remission with previous biological treatments but had developed antidrug antibodies. Exclusion criteria were concurrent participation in a clinical trial in which IBD treatment was dictated by a study protocol, prior exposure to UST, or planned cessation of treatment within 12 months for any reason. The patients were followed until withdrawal or until the pre-set study termination date as of 31 July 2021.

### Study design, follow-up, and data collection

In this retrospective, multicenter, cohort study, the patients were followed at regular outpatient visits at one of the four major teaching hospitals. Typically, patients had two to four follow-ups annually, depending on local hospital routine. Electronic medical health records were reviewed for clinical data; the index date was the day of the administration of the first intravenous infusion of UST.

A predefined standard questionnaire was developed for this study. The questionnaire included demographics, disease characteristics, drug history before-and-after UST treatment initiation, as well as clinical follow-up data, including laboratory analysis [15], weight, and endoscopy findings, when available. The data was registered into electronic case reporting forms and directly transferred into an anonymized electronic database using the Research Electronic Data Capture technique (RedCap) [16,17] hosted at Karolinska Institutet.

Physician Global Assessment (PGA) was selected for its reliance on available data types found in medical records [18]. PGA offers advantages such as flexibility in handling incomplete data and the capacity to reflect the subjective clinical judgment of treating physicians. These attributes make PGA a practical and efficient choice for retrospective analysis, contrasting with the Mayo score which necessitates endoscopic evaluation. PGA is also the most frequently used scale for retrospective studies.

### Data collection

The data was collected at fixed time intervals; at baseline, 3, 6, 9, 12, 24, and 36 months when available. Observations between these time points were carried forward to the next scheduled time point, applying the last observation carried forward techniques. Data was collected in patients with ongoing treatment through 31 July 2021. When treatment was ongoing on 31 July 2021, data on that date was classified as the last follow-up on UST. Primary outcome measures were clinical steroid-free response and remission at 3, 6, 12, 24, and 36 months, with primary focus on 3 and 12 months.

### Statistical analysis

Routine descriptive statistics were used, where the included variables were summarized as count data, percentage, or mean (minimum, maximum), SD, median, interquartile range, and proportions (%) of categorical valuables/outcomes, as proportions (%).

The main analysis was performed following the intention-to-treat principle. Discontinuation of UST was classified as treatment failure. Continuous variables were reported as median and interquartile range and censoring of distributions and categorical valuables were reported as proportions (%).

The proportion of patients with response, clinical remission, and steroid-free remission at different time points was assumed to follow a binomial distribution and 95% confidence intervals was calculated. Group comparisons were tested with the Kruskal–Wallis test.

Differences between groups were assessed using the chi-square test for trend. At all assessment time points, only patients with continued UST usage were included in the analysis. Cox regression models were applied where indicated for any relevant discrete outcome. Multivariable logistic regression was used to evaluate predictors of clinical remission at week 52; stratified by diagnosis. The survival plot for discontinuation of UST was assessed by Kaplan–Meier analysis.

The α level in the analyses was set at 5%. All tests were two-tailed and *P*-values of less than 0.05 were considered statistically significant.

## Results

### Patient characteristics

The 96 patients had a mean age of IBD diagnosis of 25, with a mean age of 36 at UST start. There were 52 males (54%) and 44 females (46%). The mean weight was 78 kg. Disease location was extensive colitis (69%), followed by left-sided colitis (29%) and proctitis (3%), (Table 1). Three months before the start of UST, 30 patients (35%) had had a colonoscopy, 29 patients (28%) had had a computed tomography scan of the abdomen and five patients (6%) had had magnetic resonance imaging to assess the severity of the disease. Drug history before the start of UST was explored. All but two patients were anti-TNF-experienced; 94 (98%) had failed ≥1, 58 (61%) ≥ 2, and three (35%) had failed ≥ 3 anti-TNF drugs (Table 1).

**Table 1.** Patient characteristics and medical history at baseline

Patient characteristics at baseline	*N* = 96, *n*/*N* (%)
Sex	
Women	44
Men	52
Mean height (cm)	174
Mean weight (kg)	78
Age at IBD diagnosis (mean) (years)	25
Age at ustekinumab treatment (mean) (years)	36
Previously fistulizing	4/96 (4.2)
History of treatment failure with biologics	
Failed ≥1	94/96 (98)
Failed ≥2	58/96 (61)
Failed ≥3	33/96 (35)
Medications for ulcerative colitis taken at baseline	
Corticosteroids orally	35/96 (36)
Corticosteroids locally	15/96 (15)
Tiopurines	13/96 (14)
Mesalazine orally	56/96 (58)
Mesalazine locally	13/96 (14)
Extension of inflammation	
Extensive colitis	65/96 (69)
Left-sided colitis	28/96 (29)
Proctitis <15 cm	3/96 (3.1)

IBD, inflammatory bowel disease.

**Fig. 1.**
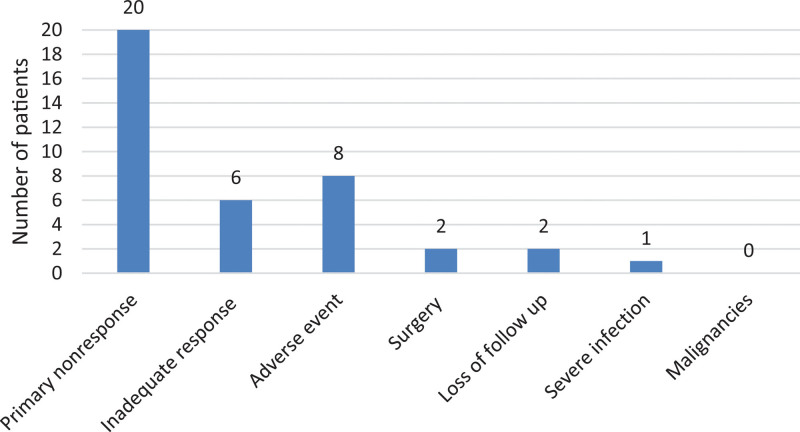
Number of patients that had to discontinue ustekinumab treatment for different reasons during the study.

In all, 54 patients (64%) were treated with mesalazine orally at the start of UST, and 13 patients (15%) were taking mesalazine locally. Furthermore, 34 patients (35%) were taking corticosteroids orally at the start of the treatment, and 14 patients (15%) were on local corticosteroids. The major reasons for discontinuing biological UST treatments (Fig. 1) were primary nonresponse (20%), adverse events (8%), and loss of response (6%).

**Fig. 2.**
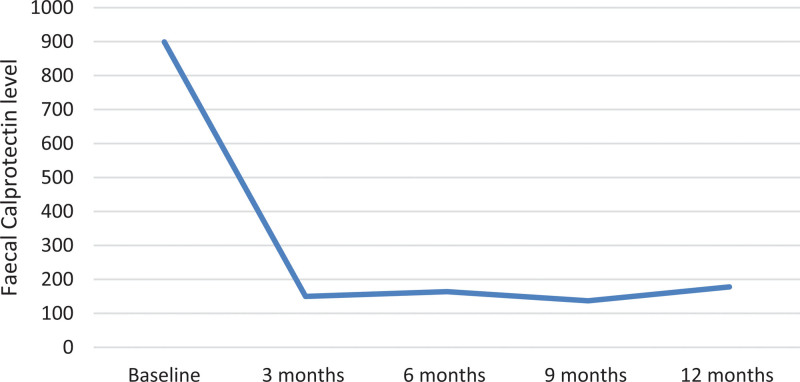
Fecal calprotectin as a marker for disease activity. The figure shows the calprotectin levels during the first 12 months of the study, with a clear reduction in calprotectin after starting treatment with ustekinumab.

The median fecal calprotectin level declined from 864 µg/g at baseline to 178 µg/g at 12 months (*P* < 0.01) (Fig. 2). C-reactive protein was stable from baseline to 12 months with a median value of 4 mg/L. A slight increase in hemoglobin and serum albumin was noted during the treatment.

### Clinical remission and effectiveness

**Fig. 3.**
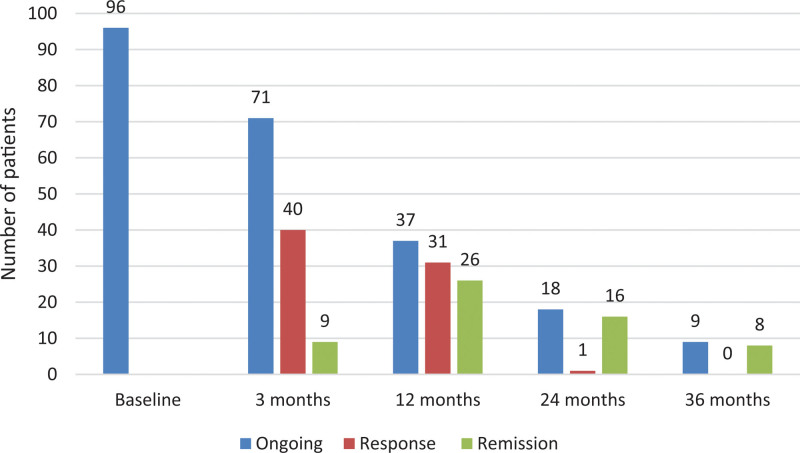
Comparison of the ongoing treatment with ustekinumab, response, and clinical remission. The comparison is between the start of the study – baseline, at 3, 12, 24, and 36 months.

The 3 months of follow-up showed that 71 patients (74%) were still on UST of which nine patients were in remission. After 6 months, 42 patients (44%) were being treated with UST of which 25 patients were in remission. At 12 months of follow-up, of 37 patients on UST treatment, 28 were in remission (Fig. 3).

**Fig. 4.**
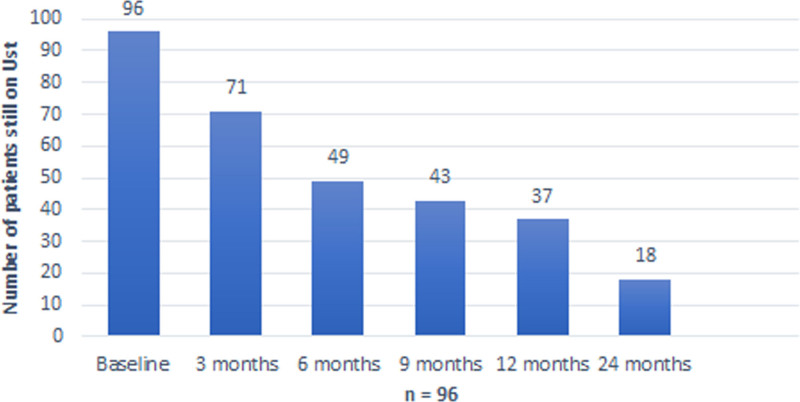
The overall drug survival plot for all ustekinumab patients, shows the number of patients treated with ustekinumab during the first 24 months of the study.

After 24 months of treatment, 19 patients were still on UST (Fig. 4). Reasons for withdrawal after 12 months were mainly no effect (3/19), loss of response (1/19), and primary nonresponsive (1/19). During the observation up to 24 months, not a single patient underwent operation.

**Fig. 5.**
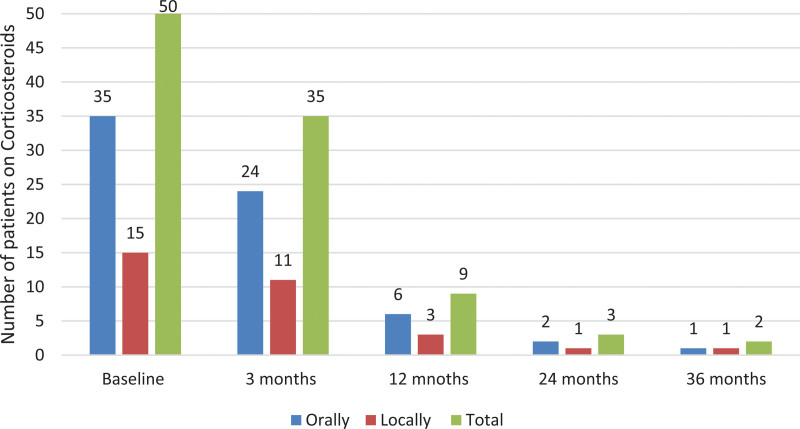
Number of patients on local and oral corticosteroids at baseline, compared with 3, 12, 24, and 36 months.

In regard to corticosteroids, at the start of the study 50 patients were on corticosteroids, orally (35 patients) and locally (15 patients). At 3 months of follow-up, 35 patients were treated with corticosteroids (combined treatment, orally 24 patients and locally 11 patients), and at 12 months, nine patients (combined treatment, orally six patients and locally three patients) were treated with corticosteroids (Fig. 5). The steroid-free remission rate is 9.4% at 3 months and 29.2% at 12 months, reflecting a significant increase in the proportion of patients maintaining remission without steroid use over time.

Initially, the majority of patients (68%) had extensive colitis, as shown in Table 1. Following treatment with UST, the proportion of patients with extensive colitis decreased to 52% at the 3-month follow-up and further declined to 27% at the 12-month follow-up. This suggests that the treatment led to a substantial reduction in the prevalence of extensive colitis among the patients over the course of a year. The chi-squared test reveals that there was no significance between left-sided colitis and extensive colitis in regard to response to UST. Therefore, knowing the phenotype of the disease has no effect on the response to the treatment. We could therefore not identify any difference between response, reason for withdrawal, and phenotype during the study.

### Endoscopic evaluation

At baseline, 33 patients (34%) underwent colonoscopy; the median value of Ulcerative Colitis Endoscopic Index of Severity (UCEIS) was 5 (interquartile range: 4–6). The limited number of follow-up endoscopies was too low (*n* = 2) and did not allow for meaningful comparison over time.

### Dosage interval

The analysis of UST dosage intervals reveals a predominant use of the 8-week interval. At 3 months, 53 patients were on an 8-week dosing schedule, 30 patients on a 12-week schedule, and only one patient each on 4- and 6-week schedules. By 12 months, the number of patients on an 8-week interval had decreased to 24, with only five patients on a 12-week interval, and a significant amount of withdrawals (47 patients). At 24 months, the 8-week interval remained the most common, with 14 patients, while missing data increased to 64 patients.

### Safety

The major reason for discontinuation was primary nonresponse (20%), followed by adverse events or intolerance (8%), loss of response (6%), need for surgery (2%), and loss of follow-up (2%). There were no reports of death or malignancy.

### Outcome 12 months after ustekinumab discontinuation

Data was collected over the 12 months following discontinuation of UST, irrespective of reason, in 20 patients. In all, 35% (7/20) started vedolizumab, 25% (5/20) started infliximab and adalimumab, respectively, 10% (2/20) started certolizumab and finally, 5% (1/20) received golimumab.

## Discussion

In this retrospective cohort study, including patients treated with UST at four teaching hospitals in Stockholm, a relatively high UST persistence, both after 3 months 74% and after 12 months 39%, were observed. The observed persistence rates were supported by improvements in F-calprotectin levels and the decline of treatment with steroids. At 12 months of UST treatment, only nine patients were still on oral or local steroids (Fig. 5) compared with nearly half of the patients (48%) at baseline. The treatment was overall well tolerated has been shown in previous studies [19,20], with primary nonresponse being the major reason to discontinue UST (20%).

Several previous studies have shown similar results as our study [21–23]. Chaparro and colleagues included 95 patients treated with UST. Overall, 33% were on remission after 12 months with the main reason to discontinue the treatment being primary nonresponse [24]. Another study in Italy, including patients with UC, showed 50% steroid-free remission after a year and 82% clinical response with UST [25]. These findings are comparable with previous studies of UST treatment in UC.

Both the current study and Thunberg *et al.*’s [20] study significantly contribute to the evolving understanding of UST in treating UC. While both studies include a substantial number of UST-treated UC patients, they provide complementary insights due to differences in study design, duration of follow-up, and specific outcome measures evaluated.

The current study, encompassing all UST-treated patients across four major hospitals in Stockholm, Sweden, provides an extensive follow-up period from UST approval to July 2021. This longer term observation allows for a comprehensive assessment of UST’s sustained efficacy and safety profile over time, essential for understanding its role in managing chronic UC and potentially influencing treatment strategies in real-world clinical practice [22–24].

Key strengths of our study include its focus on important clinical endpoints such as steroid-free remission rates and safety profiles. By evaluating these outcomes, the study underscores UST’s ability not only to induce clinical remission but also to reduce dependence on corticosteroids, thereby improving long-term disease management and patient quality of life.

In clinical practice, the rate of endoscopic examinations is often lower than in clinical trials because of patient preference [18,25,26]. Therefore, proxies of endoscopic activity, such as F-calprotectin, are often used to assess disease activity in clinical practice. We noted a low frequency of endoscopic procedures performed on patients during the observational period (*n* = 2). One possible explanation is that patients with moderate-to-severe UC in this cohort had undergone multiple endoscopies before starting UST, potentially contributing to a reduced inclination for additional examinations. In addition, the study coincided with the coronavirus disease 2019 pandemic, which likely influenced a preference to refrain from endoscopy and utilize alternative methods for assessing remission. However, data on F-calprotectin at baseline and at last follow-up were available for most of the patients and showed an improvement of F-calprotectin and albumin levels during treatment with UST (Fig. 2). Improvement in those biomarkers is a strong indication of the effectiveness of UST and the healing of the intestinal mucosa [20].

The observed proportion of patients reaching a clinical response to UST was 55% at 12 months, with primary failure as the main reason for UST discontinuation. This clinical observation and results are similar to those previously reported in clinical studies [27–29].

In our analysis of various factors, including age, sex, disease localization, and the number of previous biological drugs, we found no significant differences in outcomes or rates of remission. This finding underscores the robustness of UST as a treatment option, illustrating its efficacy across diverse demographics and disease presentations. It highlights UST’s potential advantage over therapies that may be more susceptible to variability influenced by these factors, thereby affirming its suitability for patients who have previously received multiple biological treatments. Importantly, even the number of previous biological drugs did not alter the treatment outcome, further supporting UST’s consistent therapeutic benefit in challenging cases.

The analysis of the dosage interval reveals that the general decline in patient numbers for longer intervals over time does not correspond with an increase in the use of shorter intervals. This indicates that the overall dosing interval for UST does not trend towards shorter periods over time.

The data analysis did not clearly demonstrate any benefit from shortening the UST dosing intervals. The findings imply that adjusting the interval did not lead to a higher remission rate.

The study limitations were the timing of colonoscopies and the small number of patients that underwent colonoscopy during follow-up. This limits the conclusion to clinical response, steroid-free response, and clinical remission.

### Conclusion

UST was found to be an effective biological drug in difficult-to-treat patients, with an overall favorable safety profile and a high probability of reaching remission and inducing a corticosteroid-free stage after 12 months. The overall improvement in PGA adds to the observed effectiveness of the drug as also was shown with the reduction in F-calprotectin.

## Acknowledgements

This work was supported by Janssen Cilag AB as a company-sponsored study. The sponsor and its employee had no access to the data at any time or any role in the data management and data analysis of the study.

Guarantor of the article: C.S. Conception and design of the study: S.A., M.L., C.S., O.F., and C.W. Generation, collection, assembly, and interpretation of data: H.S., F.B., S.M., A.B., F.J., C.H., O.F., C.W., C.S., M.L., and S.A. Statistical analyses: F.J., H.S., and C.S. Drafting of the manuscript: H.S. and C.S. Revision of the manuscript: H.S,. F.J., C.H., O.F., C.W., S.A., and C.S. Approval of the final version of the manuscript: H.S., F.B., A.B., S.M., F.J., C.H., O.F., C.W., M.L., S.A., and C.S.

The study was approved by the Institutional Review Board, ‘Etikprövningsmyndigheten’, Stockholm, on 19 February 2020 (Dnr 2020-00019), and, through an amendment covering the three other hospitals on 16 May 2021 (Dnr 2021-00360). The requirement for informed consent to participate was waived by the institutional review board.

### Conflicts of interest

H.S., F.B., and A.B. performed part of the work during their internship in gastroenterology at Region Stockholm. H.S.: speaker: Janssen and research: Janssen. CH: speaker: Janssen, Vifor-Pharma, Takeda. O.F. and C.W. are employees of Janssen Cilag AB. S.A.: scientific committee/advisory board: Pharmacosmos, Janssen, Takeda; consultant: Takeda, Janssen; speaker: Galapagos, Janssen, Tillotts; and research: Janssen. For the remaining authors, there are no conflicts of interest.
